# Efficient Workload Classification based on Ignored Auditory Probes: A Proof of Concept

**DOI:** 10.3389/fnhum.2016.00519

**Published:** 2016-10-13

**Authors:** Raphaëlle N. Roy, Stéphane Bonnet, Sylvie Charbonnier, Aurélie Campagne

**Affiliations:** ^1^Université Grenoble AlpesGrenoble, France; ^2^Gipsa-Lab, Centre National de la Recherche ScientifiqueGrenoble, France; ^3^CEA LETIGrenoble, France; ^4^Laboratoire de Psychologie et NeuroCognition, Centre National de la Recherche ScientifiqueGrenoble, France

**Keywords:** workload, classification, auditory evoked potentials, spatial filtering

## Abstract

Mental workload is a mental state that is currently one of the main research focuses in neuroergonomics. It can notably be estimated using measurements in electroencephalography (EEG), a method that allows for direct mental state assessment. Auditory probes can be used to elicit event-related potentials (ERPs) that are modulated by workload. Although, some papers do report ERP modulations due to workload using attended or ignored probes, to our knowledge there is no literature regarding effective workload classification based on ignored auditory probes. In this paper, in order to efficiently estimate workload, we advocate for the use of such ignored auditory probes in a single-stimulus paradigm and a signal processing chain that includes a spatial filtering step. The effectiveness of this approach is demonstrated on data acquired from participants that performed the Multi-Attribute Task Battery – II. They carried out this task during two 10-min blocks. Each block corresponded to a workload condition that was pseudorandomly assigned. The easy condition consisted of two monitoring tasks performed in parallel, and the difficult one consisted of those two tasks with an additional plane driving task. Infrequent auditory probes were presented during the tasks and the participants were asked to ignore them. The EEG data were denoised and the probes’ ERPs were extracted and spatially filtered using a canonical correlation analysis. Next, binary classification was performed using a Fisher LDA and a fivefold cross-validation procedure. Our method allowed for a very high estimation performance with a classification accuracy above 80% for every participant, and minimal intrusiveness thanks to the use of a single-stimulus paradigm. Therefore, this study paves the way to the efficient use of ERPs for mental state monitoring in close to real-life settings and contributes toward the development of adaptive user interfaces.

## Introduction

Mental workload is frequently defined as task difficulty and the associated mental effort (Gevins and Smith, 2007). It is therefore of critical interest to better assess this state to the human factor community who aims at developing smart technologies that enhance operator’s safety and performance. The impact of workload on behavior has been extensively documented. Participants’ reaction time is known to increase linearly with the increase in the number of items to memorize (Sternberg, 1969), as well as with the number of tasks to perform in parallel (Cain, 2007). However, behavioral responses are not always enough for mental state monitoring (MSM) systems, mainly due to their latency of occurrence, and to the fact that some mental states are not necessarily or systematically reflected by a specific response. Physiological data give more insight into the operator’s state, especially electroencephalography (EEG), a method that allows for direct mental state assessment. The use of physiological markers derived from the cerebral activity for human factor purposes has given rise to a new field: neuroergonomics (Parasuraman et al., 2012).

Amongst the various markers derived from the EEG activity, event-related potentials (ERPs) are frequently used for MSM. ERPs correspond to the EEG activity that is temporally locked to the appearance of a given stimulation, or probe. Although, ERPs only allow for a discontinuous evaluation of the operator’s mental state -unlike frequency measures, according to Roy et al. (2016) frequency measures are very sensitive to mental fatigue and vigilance states whereas ERPs are more robust to these states. Therefore, ERPs may be more suitable for ecological settings. Moreover, the literature describes numerous workload-related ERP modulations, such as early and late components’ amplitude decreases. Hence, the P300 component’s amplitude is reduced by an increase in workload (Kok, 2001; Schultheis and Jameson, 2004; Gomarus et al., 2006; Holm et al., 2009; Friedrich et al., 2011), and so is the N1, N2, and P2 components’ amplitude (Kramer et al., 1995; Ullsperger et al., 2001; Gomarus et al., 2006; Allison and Polich, 2008; Miller et al., 2011; Boonstra et al., 2013). In the specific context of simulated flight, the P300 component’s amplitude elicited by auditory probes has also been shown to decrease when the primary task’s complexity increases (Natani and Gomer, 1981; Kramer et al., 1987; Sirevaag et al., 1993).

In order to determine an operator’s mental state to modify the behavior of a system, one needs to compute an index or a class of workload to be fed as an input. This can be done using machine learning algorithms developed for brain–computer interfaces (BCIs). When those algorithms are used for applications that are not directed toward the voluntary control of an effector, those systems are often referred to as passive BCIs (Zander and Kothe, 2011; van Erp et al., 2012). Although, the number of publications regarding mental workload assessment has drastically increased this decade, only a few articles actually propose a classification based on ERPs. Brouwer et al. (2012) used seven electrodes and achieved 64% of correct binary classifications. Recently, it was proved that ERP spatial filtering could significantly enhance workload classification (Mühl et al., 2014; Roy et al., 2015). The authors achieved 72 and 98% of correct classifications using respectively a Fisher spatial filtering (FSF; Hoffmann et al., 2006) and a canonical correlation analysis filtering (CCA; Hotelling, 1936). However, these authors used task-dependent probes, i.e., items that were paramount for the task at hand, which is therefore quite unrealistic for real-life settings. Very recently, Roy et al. (2016) showed that ERPs elicited by visual task-independent probes could be used for mental workload estimation. They inserted a basic detection task in a Sternberg memory task and used the ERPs elicited by the targets to classify the workload level of the memory task. They reached 91% of correct binary classifications by filtering the ERPs using a CCA. This is very promising, however the probes, although task-independent, still required an overt answer from the participant. This kind of dual task setting can therefore lead to decreased attentional engagement to the primary task, which seems rather unwelcome for operators’ monitoring in hazardous work situations (e.g., driving, plant monitoring, custom control). Hence, the best approach to use ERPs in ecological settings would be a stimulation paradigm with task-independent and ignored probes. And as the ultimate goal should be to develop systems based on minimally intrusive probes, these stimulations should be as scarce as possible. As reported by Mertens and Polich (1997), the ERPs elicited in a single-stimulus paradigm by visual or auditory probes are a viable alternative to the traditional oddball procedure, although late components’ amplitude is reduced when the stimuli are ignored compared to when they are counted or await a motor response. The authors even report that auditory probes elicit ERPs that are more robust to response type. That is to say that ignored auditory stimuli generate early and late components which amplitude is quite similar to that of stimuli awaiting an active answer. This makes them very good candidates for the features to use in a mental workload estimation procedure.

This study intends to provide an evaluation of the efficiency of a workload estimation based on the ERPs elicited by infrequent, task-independent and ignored auditory probes. Workload was modulated by modulating the number of tasks to perform in parallel with the Multi-Attribute Task Battery – II (MATB; Comstock and Arnegard, 1992). A single-stimulus paradigm was used to elicit ERPs which were then spatially filtered with a CCA and classified. The performance of this processing chain was also compared to that of a simpler chain without spatial filtering. The contributions of this paper are threefold: (1) to assess the validity of the single-stimulus paradigm for effective mental workload estimation; (2) to assess the relevance of a processing chain that includes a spatial filtering step in order to classify accurately the auditory evoked potentials (AEPs) of those ignored, infrequent probes; (3) to assess the relevance of both the stimulation paradigm and the processing chain for an ecologically valid task, the MATB.

## Materials and Methods

This research was promoted by Grenoble’s clinical research direction (France) and was approved by the French ethics committee (ID number: 2014-A00040-47) and the French health safety agency (B140052-31).

### Experimental Setup

**FIGURE 1:**
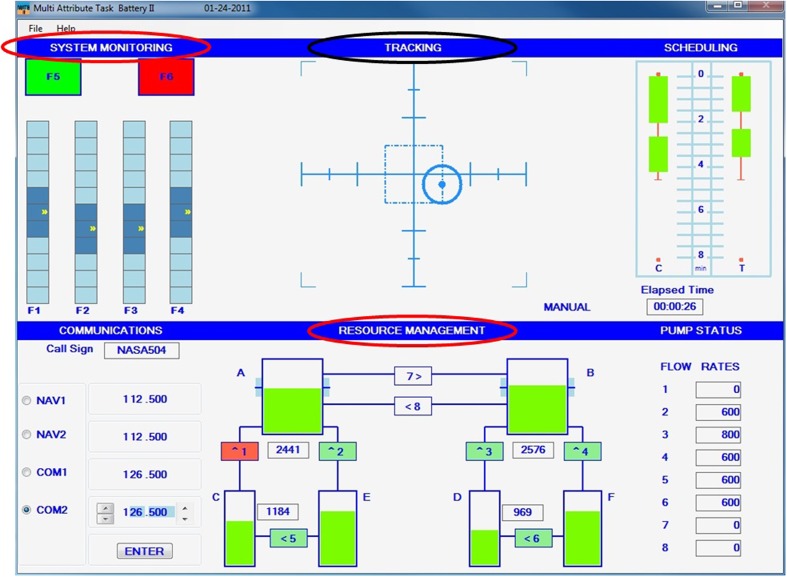
**Multi-Attribute Task Battery – II.** The participants performed two out of three of the circled sub-tasks during the low workload condition (system monitoring and resource management tasks), and an additional third task during the high workload condition (tracking task).

Eight healthy right-handed volunteers (three females; 29.9 years old ± 5.9) performed two 10-min experimental blocks of the Multi-Attribute Task Battery-II, the last version of task developed by NASA to study divided attention and multitasking (Comstock and Arnegard, 1992; **Figure 1**). In this experimental setup, each block corresponded to a different workload level (low/high), which was pseudo-randomly assigned. In the low workload condition, the participants performed two monitoring tasks using the keyboard, i.e., the system monitoring and the resource management tasks. The system monitoring task was presented in the upper left window of the display. As explained in the article of Comstock and Arnegard (1992), the demands of monitoring gages and warning lights were simulated here. The participants had to respond to the absence of the green light, the presence of the red light, and to monitor the four moving pointer dials deviation from midpoint. Regarding the resource management task, it simulated the demands of fuel management. The participants had to maintain tanks A and B at 2500 units each. This was done by turning on or off any of the eight pumps, which can sometimes fail.

In the high workload condition, they had an additional tracking task to manage in parallel. The tracking task was located in the upper middle window and simulated the demands of manual control. The participants had to keep the target at the center of the window using the joystick. Therefore, in both the low and high workload conditions perceptual, attentional, and decision making processes are recruited, along with motor preparation and performance. The difference between the low and the high workload conditions only stems in the additional workload imposed by the additional task.

**FIGURE 2:**
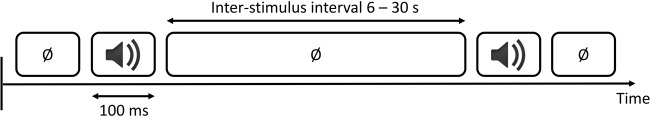
**Single-stimulus paradigm using ignored and infrequent auditory probes**.

In addition to the visual stimulations induced by the MATB-II, the participants received auditory stimuli. They were instructed to ignore these auditory stimuli and to focus on the task at hand. These stimuli were sent by the Eprime software (E-prime Psychology Software Tools, Inc., Pittsburgh, PA, USA) into their Sennheiser audioset. In a similar fashion to the single-stimulus paradigm of Allison and Polich (2008), they consisted of 100 ms 1000 Hz pure tones (10 ms rise/fall, 65 dB SPL), with a random 6–30 s inter-tone interval (**Figure 2**). A minimum of 30 stimulations per block were presented.

### Data Acquisition

Data acquisition was performed at the IRMaGe Neurophysiology facility (Grenoble, France). The participants’ answers to the Rating Scale Mental Effort questionnaire (RSME; Zijlstra, 1993) and their resource management task root mean square (RMS) error scores were recorded, as well as their EEG activity using an Acticap^®^ (Brain Products, Inc.) equipped with 32 Ag-AgCl unipolar active electrodes that were positioned according to the 10–20 system. The reference and ground electrodes used for acquisition were those of the Acticap, i.e., FCz for the reference, and AFz for the ground. The electro-oculographic activity was also recorded using two electrodes positioned at the eyes outer canthi, and two respectively above and below the left eye. Impedance was kept below 10 kΩ for all electrodes. The signal was amplified using a BrainAmp^TM^ system (Brain Products, Inc.) and sampled at 500 Hz with a 0.1 Hz high-pass filter and a 0.1 μV resolution. Participants were instructed to limit eye and body movements during the task.

### Signal Processing

**FIGURE 3:**
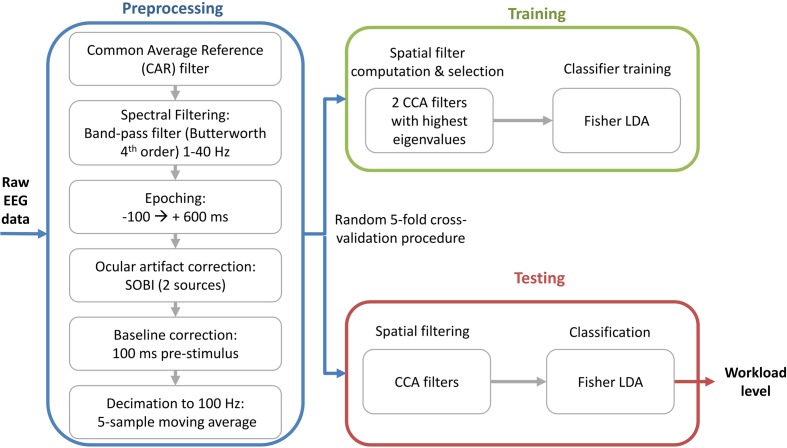
**Flow diagram of the signal processing chain applied on the EEG data in order to estimate mental workload**.

The processing chain is detailed in **Figure 3.** In a general manner, the raw data was preprocessed, then spatially filtered, and lastly classified. Details are given in the following sub-sections regarding each step of this chain. It should be noted that the same processing chain was replicated without the spatial filtering step in order to evaluate if spatial filtering enhances the discriminability of the two workload levels.

#### Preprocessing

The digital EEG signal was band-pass filtered between 1 and 40 Hz, and re-referenced to a common average reference. The signal was then epoched starting 100 ms before and ending 600 ms after the auditory stimulation. Next, artifacts related to ocular movements (saccades and blinks) were corrected using the signal recorded from the electrooculographic electrodes (EOG) and the Second Order Blind Identification algorithm (SOBI; Belouchrani et al., 1997). This algorithm was chosen to perform the source decomposition because thanks to its assumption of non-correlation –and not mutual independence- it has been shown to be more suitable for electrophysiological data by Congedo et al. (2008). In order to get closer to a system that could be implemented on-line in a real-life setting, the two sources that were the most correlated to the EOG activity were canceled. All trials were kept for analysis. The AEPs were then extracted by subtracting a 100 ms baseline (i.e., mean signal amplitude) to the 600 ms segment that starts at the onset of the stimulation. Lastly, the data was decimated to 100 Hz using a five-point moving average.

#### Spatial Filtering

Then, the preprocessed data **X** (*N_s_* – number of samples × *N_e_* – number of channels) were spatially filtered, resulting in the signal **Z = WX** (*N_s_ –* number of samples × *N_f_* – number of spatial filters). Each column of the matrix **W** contains a spatial filter with its spatial pattern in the corresponding column of **A** = (**W**^-1^)^T^. In this paper, we use CCA as a spatial filtering method. As Spüler et al. (2014) detailed it , in a two-class scenario the CCA filters are computed in order to maximize the correlation between the EEG signal **X** and the matrix **Y** = **D**_1_**P**_1_+**D**_2_**P**_2_ that contains the time replication of the average ERP responses **P**_i_ for each class. The matrix **D**_i_ is a Toeplitz binary matrix that indicates the stimulation onset for the i^th^ class (Rivet et al., 2009). Several methods have been proposed to solve CCA by computing orthonormal bases for the data matrices either by QR or singular value decomposition – SVD (Björck and Golub, 1973).

The CCA spatial filters were computed using the training data only. Then, the spatial filters with the two highest associated canonical correlations were selected. When these filters are applied on the testing data, the feature vector for the j^th^ trial is given by the column concatenation **f**_j_ = vec(X_j_[**w**_1_**w**_2_]) with dimension 120x1 (i.e., 60 samples × 2 virtual electrodes). In order to have the same number of features for both processing chains (with and without spatial filtering), for the chain without spatial filtering the feature vector was composed of the concatenated signals of the C3 and Pz electrodes (chosen visually using the average spatial patterns presented in Section “Spatial patterns”).

#### Classification

A single-trial classification was performed on the feature vector **f** using a Fisher linear discriminant analysis (FLDA), with a shrinkage estimation of the covariance matrices (Schäfer and Strimmer, 2005). As explained by Blankertz et al. (2011), this estimation method allows the use of LDA with high dimensional features and gives good results that can generalize well (Blankertz et al., 2011). We used a random fivefold cross-validation procedure. The spatial filters were learned on the training set, and applied on the testing set. In the same way, the shrinkage estimation was learned on the training set. The performance of the processing chains was assessed based on their intra-subject binary classification accuracy.

### Statistical Analyses

Statistical analyses were carried out on all results, i.e., subjective results from the RSME questionnaire, N1, P1, N2, P2, and P3 peak amplitude and latency from the AEP components, and classification results obtained using the processing chains with and without spatial filtering. All results were compared between themselves using repeated measures ANOVAs and Tukey *post hoc* tests. The significance level was set at 0.05.

## Results

### Behavioral and Subjective Data

In a similar manner to Fournier et al. (1999), behavioral responses were standardized within each participant by dividing their response times to the resource management tasks by their proportion of correct responses. There was a significant effect of workload on this performance score (*t* = 2.99, *p* < 0.05), the participants’ performance was significantly degraded in the high workload condition compared to the low workload condition (m1_perf = 0.33; sd1_perf = 0.12; m2_perf = 0.43; sd2_perf = 0.12). Moreover, the participants reported having furnished a significantly bigger effort in the high workload condition than in the low workload one [*F*(1,7) = 38.04, *p* < 0.01; m1_RSME = 45.5; sd1_RSME = 18.2; m2_RSME = 71.6; sd2_RSME = 24.3].

### Auditory Evoked Potentials

**FIGURE 4:**
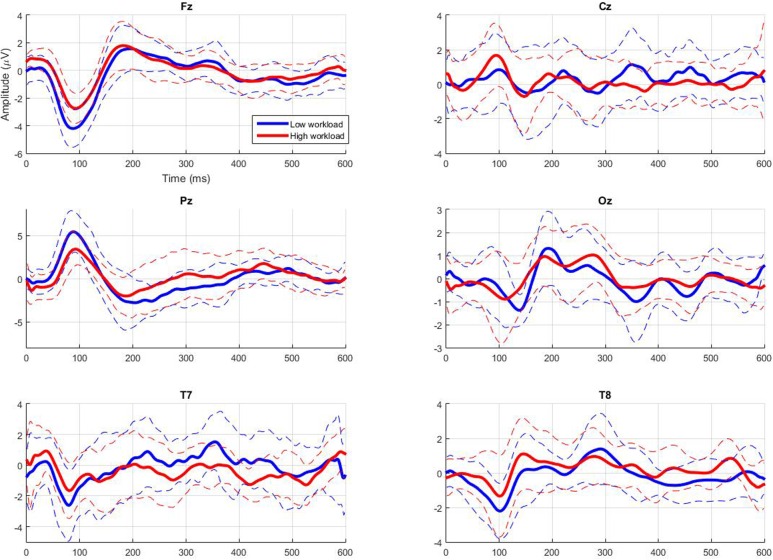
**Grand average (in bold) and standard deviation (dotted line) of the auditory evoked potentials (AEPs) elicited by the ignored infrequent auditory probes depending on workload condition at major midline electrode sites (Fz, Cz, Pz, and Oz), as well as at auditory processing relevant sites (T7 and T8)**.

**FIGURE 5:**
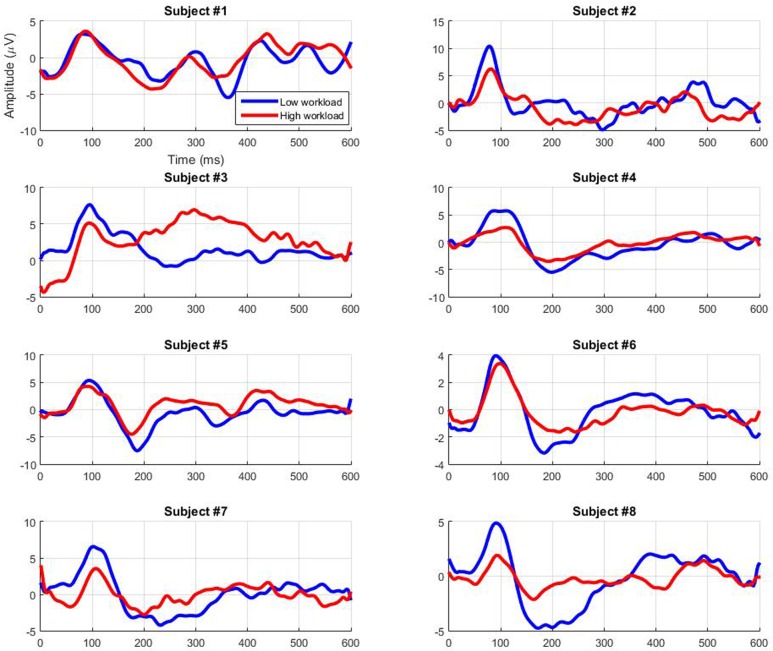
**Auditory evoked potentials elicited by the ignored infrequent auditory probes depending on workload condition at the Pz electrode site for all participants (grand average across trials)**.

**Figure 4** gives the grand-average AEPs across participants at major median electrode sites (Fz, Cz, Pz, and Oz), as well as at electrode sites located close to the auditory cortex (T7 and T8). **Figure 5** also gives the individual AEPs for the eight participants at the Pz electrode site (chosen to illustrate the results that follow regarding early components). The typical components reported to be modulated by workload can be noticed, i.e., N1, P1, P2, N2, and P3 (Kramer et al., 1995; Kok, 2001; Ullsperger et al., 2001; Schultheis and Jameson, 2004; Gomarus et al., 2006; Allison and Polich, 2008; Holm et al., 2009; Miller et al., 2011; Boonstra et al., 2013). However, the statistical analyses revealed only few significant results at the group level, which is understandable given the mostly overlapping variance of both signals (see standard deviations in **Figure 4**). Indeed, with increasing workload there were only trends at the Pz electrode for a decrease in amplitude of the P1 component (*p* = 0.11; **Figure 5**) and for a decrease in latency of the N1 component (*p* = 0.07). Moreover, when workload increased there was a significant decrease in latency of the P2 component at all electrode sites [*F*(1,7) = 6.74, *p* < 0.05].

### Spatial Patterns

**FIGURE 6:**
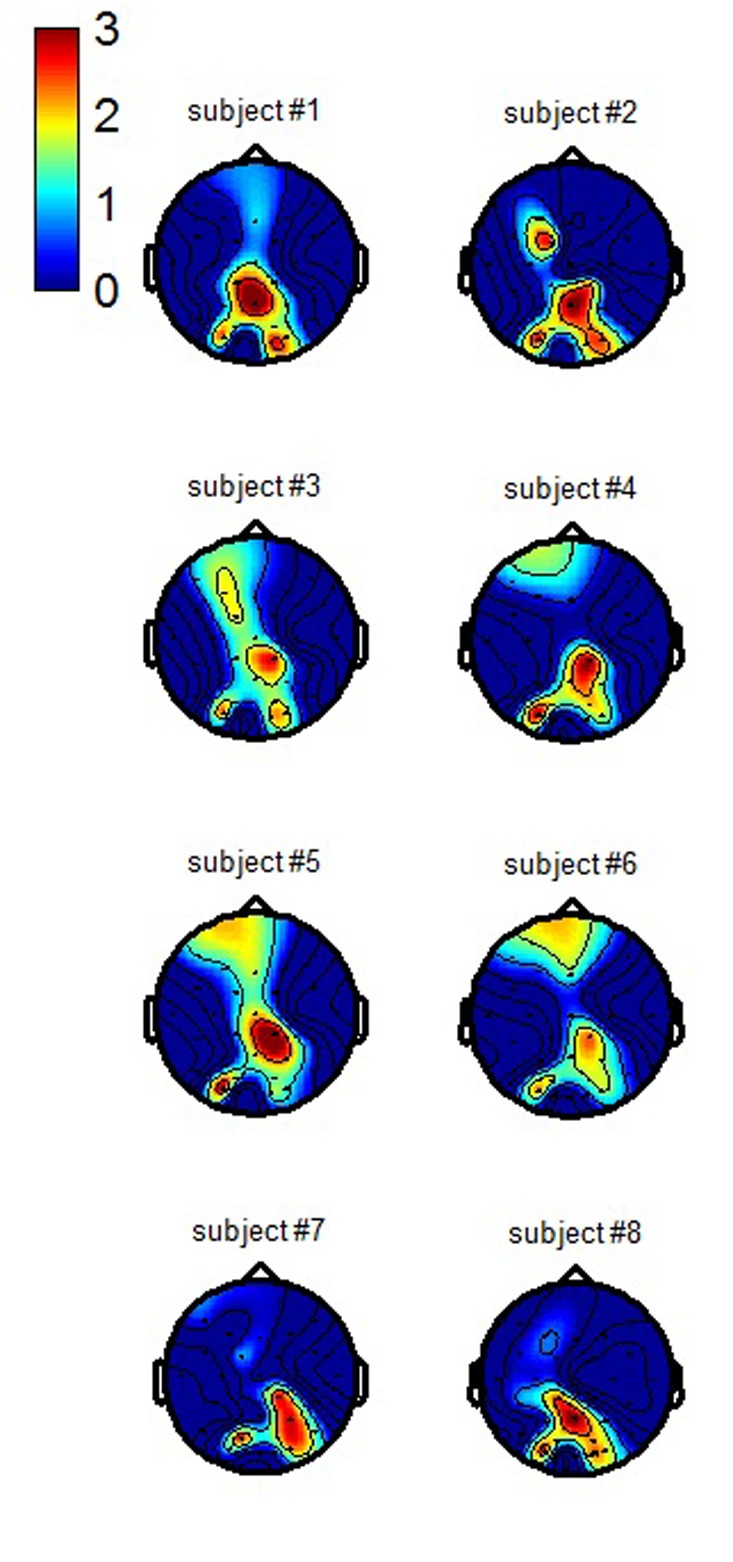
**Individual CCA spatial patterns of the first filter used to enhance the discrimination of the workload condition (grand average across training folds)**.

**FIGURE 7:**
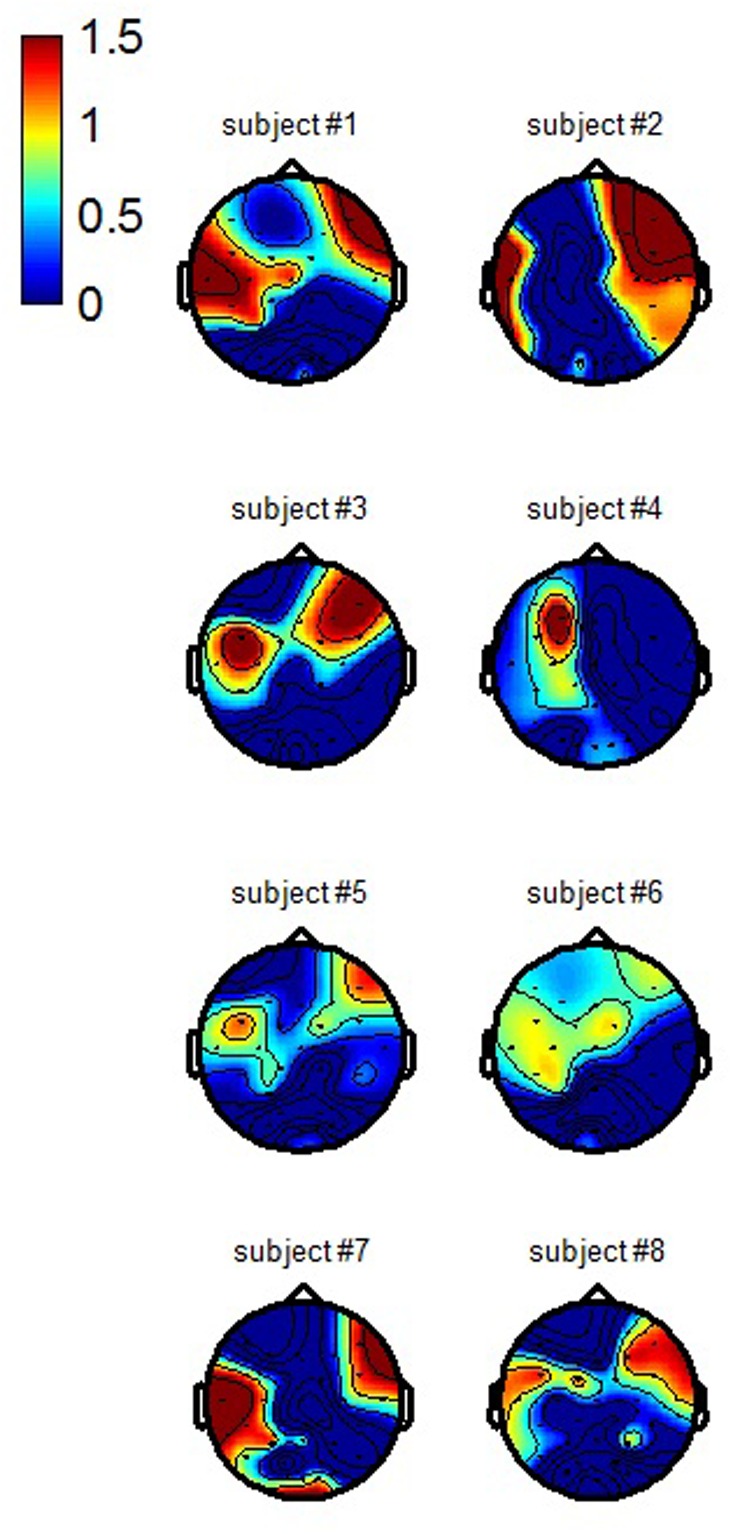
**Individual CCA spatial patterns of the second filter used to enhance the discrimination of the workload condition (grand average across training folds)**.

The topographical representation of each of the two CCA spatial patterns obtained for each participant using the processing chain proposed in this paper are presented respectively in **Figures 6** and **7** (average across training folds). The first spatial pattern reveals that in order to better discriminate workload levels, our first selected spatial filter enhances the activity from centro-parieto-occipital regions -consistent with attentional processing, while the second one enhances the activity from temporal regions -consistent with auditory processing- as well as prefrontal areas which could be related to ocular activity.

### Filtered EEG Signal

**FIGURE 8:**
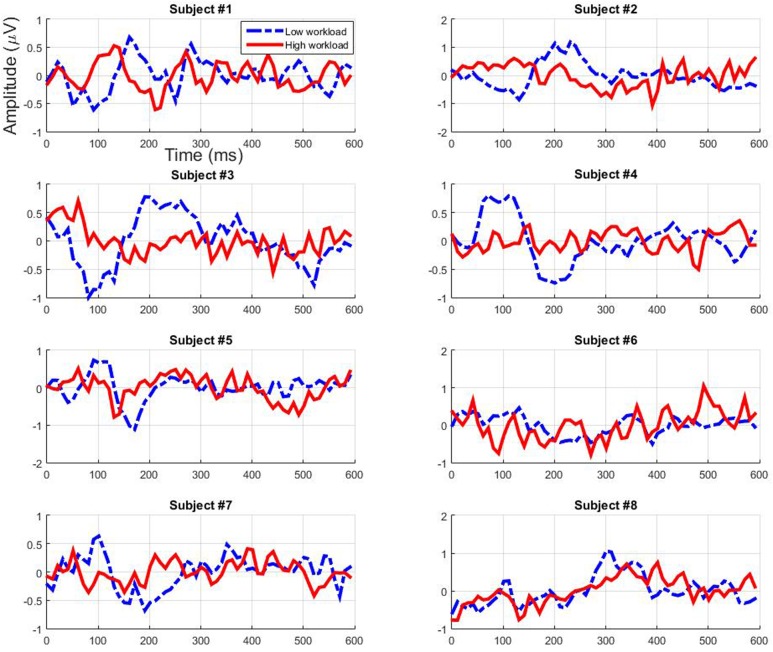
**Individual filtered test data – using the first CCA filter – depending on workload condition (grand average across cross-validation folds)**.

**FIGURE 9:**
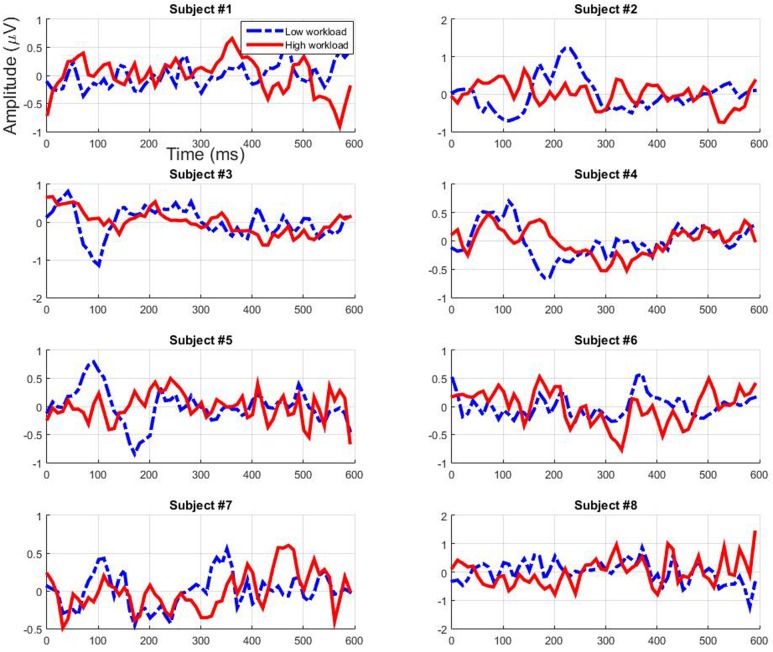
**Individual filtered test data – using the second CCA filter – depending on workload condition (grand average across cross-validation folds)**.

The filtered EEG signals obtained from the testing sets for each participant using the first and second CCA filters are presented respectively in **Figures 8** and **9** (grand average across cross-validation folds). Both filters seem to mainly enhance the ERP activity of the low workload condition while decreasing it for the high workload condition from around 50 to 400 ms. This is particularly true for participants 3 and 4 for the early components. The signal’s polarity fluctuates in a different manner depending on the participant and the filter, however, a general pattern emerges. Particularly, for both filters we can see an enhancement in the low workload condition of the amplitude of the early components that peak between 80 and 250 ms, be it in the negative or in the positive range. Therefore it seems that the filters act in a way so that they enhance the relevance of early auditory evoked components but not so much of later components.

### Classification Accuracy

**FIGURE 10:**
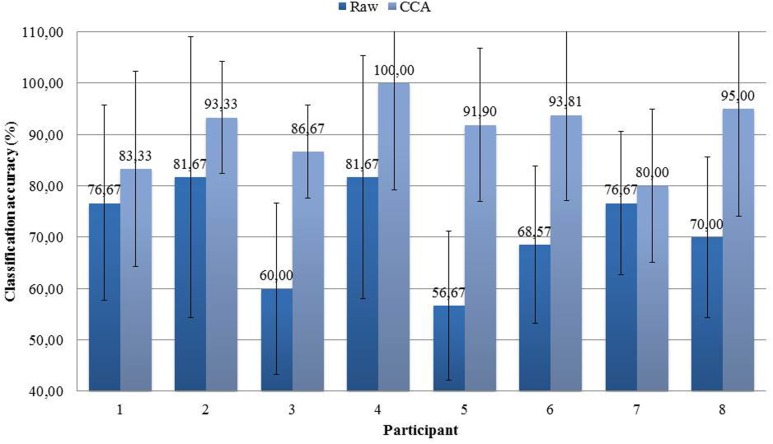
**Workload level classification accuracy reached for each participant using the raw data of two electrodes or a CCA spatial filtering step (mean and standard deviation of the percentage of correct binary classification across the random fivefold cross-validation procedure)**.

The workload level classification results obtained using the single-stimulus paradigm and both the processing chain that includes a CCA spatial filtering step and the simpler processing chain without spatial filtering are given by **Figure 10** for each participant. There was a significant effect of the type of processing chain [*F*(1,7) = 39.90, *p* < 0.001]. Indeed, the chain that included the CCA spatial filtering step gave higher classification results than the one that didn’t. The mean percentage of correct binary classification across the eight participants was 90.51% (± 10.7 SD) and 71.49% (± 15.9 SD) respectively for the processing chains with and without spatial filtering. Using the chain that included the CCA filtering, the performance was optimal for participant 4 with a classification accuracy of 100% and a null standard deviation, and the lowest performance was obtained for participant 7 with a classification accuracy of 80% and a very large standard deviation of 21.73.

## Discussion

Studies have demonstrated that workload modulates the ERPs elicited by attended or ignored auditory probes in a classical oddball paradigm involving deviant and standard tones (Kramer et al., 1995). Allison and Polich (2008) had also demonstrated this phenomenon using only infrequent standard tones (i.e., single-stimulus paradigm). However, to our knowledge, there was no literature regarding effective workload *classification* based on ignored auditory probes. Indeed, no signal processing chain had been applied to estimate workload in an automatic way from the ERPs of ignored auditory stimuli. Hence, this study was intended to bring new light on the potential use of ignored infrequent task-independent probes to efficiently and *automatically* assess mental workload in a minimally intrusive way. In order to do so, a single-stimulus paradigm similar to that of Allison and Polich (2008) was used, along with a processing chain that included a CCA spatial filtering step. The participants rated their effort as significantly higher for the high workload condition than for the low one and also exhibited a decrease in performance in the high workload condition compared to the low workload condition akin to that observed by Fournier et al. (1999). Their ERPs revealed only trends for a decrease in P1 amplitude and N1 latency, as well as a significant decrease in P2 latency. These results are in line with the literature regarding resource allocation processes. In a general manner, the amplitude of the ERP components that occur within the first 250 ms following stimulus onset has been demonstrated to be influenced by attentional capacity allocated to the eliciting stimulus and task operations (for a review see Kok, 1997). For instance, the P1 component amplitude is larger in active relative to passive viewing conditions (Fu et al., 2010). As for the N1 and the P2 latency, it has also been shown to decrease with a decrease in allocated attentional resources (Okita, 1979; Callaway and Halliday, 1982). However, more differences in amplitude were expected based on articles that describe workload modulations for ERPs elicited by task-dependent stimuli, specifically on late ERP components’ amplitude (Kok, 2001; Ullsperger et al., 2001; Schultheis and Jameson, 2004; Gomarus et al., 2006; Holm et al., 2009; Miller et al., 2011; Boonstra et al., 2013). Yet, Kramer et al. (1995) had found that only the early components of the ERPs elicited by ignored task-irrelevant probes were relevant to perform a non-intrusive workload assessment, and that the late P300 component was not a good marker for such a goal. Our results confirm theirs as to which components are significantly modulated by workload using ignored auditory probes.

Despite the few significant results obtained at the group level regarding AEP components’ amplitude, very accurate mental workload estimations were obtained using a signal processing chain that included a CCA spatial filtering step with at least 80% of correct binary classification accuracy for all participants, and an average of 90.51%. This result is in line with the literature that shows that classifiers can reveal statistical differences when standard statistical tests between ERPs do not (Noh and de Sa, 2014). Also, here the use of the CCA spatial filtering step significantly enhanced the estimation performance, as already demonstrated by Roy et al. (2015), and also reduced the variance in the results. Besides, this is only slightly lower than what Roy et al. (2016) obtained using task-independent visual probes -91%. This is very promising given that here, opposite to their protocol, the task-independent probes required no overt response and were ignored by the participants. Moreover, the probes used in this experiment are auditory while they were visual in their protocol. Lastly, those results are also higher than that obtained by previous studies that classified raw or spatially filtered ERPs elicited by task-dependent probes (Brouwer et al., 2012; Mühl et al., 2014). Therefore, the use of the single-stimulus paradigm coupled to a processing chain that includes a spatial filtering step allows a precise estimation of mental workload for a task that is very close to an actual work task. A limitation to this study is the number of trials, although in ecological settings it will be difficult to use more probes and to remain minimally intrusive. Nevertheless, according to Combrisson and Jerbi (2015), if we have more than 20 trials our performance should be over 70% in order to account for a significant detection with a *p* < 0.05 significance rate. Here, using the spatial filtering step we obtained at least 80% of correct detections, and an average of 90.51% with a minimum of 30 trials per condition. Therefore, we can say that our results were significantly above chance and that our method is quite efficient.

The spatial patterns of the selected CCA filters revealed that an enhancement of temporal and centro-parietal activity allowed reaching such high classification results. This is in accordance with the auditory nature of our probes. It is interesting to note that the activity that was enhanced by the spatial filters in the previously mentioned study of Roy et al. (2016) who used task-independent visual probes originated from the occipital sites, in accordance with the visual nature of their stimuli. In our study, given that the probes were auditory, we observed a specific enhancement of the activity from the temporal electrode sites. The signal from the centro-parietal sites was also enhanced in their study as in ours. These sites are known to be involved with attentional processing, and more generally with resource engagement (Kok, 1997). Additionally, the patterns also revealed an implication of prefrontal sites, which could stem from an under-efficient ocular artifact correction step in our processing chain. Indeed, in order to preserve the cerebral activity as much as possible, we only deleted the 2 out of 32 sources that were the most correlated to respectively the vertical and horizontal EOG channels. Also, given that the MATB-II is a task that is very close to a real work task, it elicits more ocular movements than classical laboratory tasks during which participants are asked to fixate the center of the screen and to limit eye movements and blinks. In any case, if it is indeed ocular activity that our second spatial filter enhanced, it means that this ocular activity allows efficient mental workload estimation. This is not surprising given that blink frequency has been reported to vary depending on task difficulty (Holland and Tarlow, 1972; Tanaka and Yamaoka, 1993). Moreover, it is known that the appearance of an unexpected stimulation leads to a startle eyeblink reflex. This reflex is attenuated during a multiple-task –high workload- compared to a single-task condition –low workload (Neumann, 2002). Therefore, the ocular activity produced in response to an infrequent auditory probe could be an efficient marker of task engagement and mental workload. As Roy et al. (2014) already argued , if ocular activity is helping to discriminate workload levels, why remove it? Hence, it might be interesting for future developments to use a processing chain that either does not include an ocular artifact correction step, or, that does but performs classification by fusing two feature vectors, a clean EEG one and an ocular activity one. Multimodality in terms of origin of the physiological markers (e.g., cerebral or ocular) could therefore be the key to enhance classification accuracy for real-life implementations.

Besides, this study evaluates the relevance of a stimulation paradigm and its dedicated processing chain for an ecological task which is the MATB. Although still in a laboratory setting, this task is very close to that performed by pilots and air traffic controllers. However, it modulates workload only by varying the number of tasks to perform in parallel, that is to say by varying the participants’ degree of divided attention. In order to pursue the evaluation of the relevance of this stimulation paradigm, future work should focus on an evaluation of its relevance for several tasks that modulate workload based on different cognitive functions, e.g., working memory load, divided attention, executive functions. To our knowledge, only Berka et al. (2007) assessed the relevance of an EEG marker across several types of tasks. But, they focused on frequency power in the classical EEG bands. Thus, in order to progress toward an efficient estimation in real-life settings, the literature still lacks a thorough comparison of ERP modulations due to workload across several tasks. Also, although the participants of our study told us that they were not annoyed by the auditory infrequent stimulations and generally entirely forgot about it, a more thorough investigation of the real cost of such a paradigm in terms of operator fatigue and efficiency should be carried out. What’s more, in order to increase the practicality of EEG measures, the number of electrodes should be diminished. However, this study, along with that of Roy et al. (2015) clearly establishes the relevance of a spatial filtering step in order to enhance the discriminability between the two workload levels. Therefore, future studies should evaluate how to reduce the number of electrodes while keeping enough channels to efficiently apply such a filtering step.

Consequently, this study contributes to the neuroergonomics research topic on mental workload estimation by uncovering three main points. First, the single-stimulus paradigm in which participants are probed by infrequent task-independent and ignored probes allows minimally intrusive workload estimation. Second, a spatial filtering step such as a CCA filtering enables a very accurate AEP-based workload classification. Lastly, the combination of this single-stimulus paradigm with infrequent ignored probes and its dedicated processing chain allows efficient workload estimation for an ecologically valid task such as the MATB.

## Conclusion

This study has demonstrated as a proof-of-concept that a single-stimulus paradigm based on infrequent ignored auditory probes and its dedicated processing chain could allow a very accurate estimation of mental workload with a classification performance above 80% for every participant. This is also the first study to effectively classify workload based on ERPs elicited by ignored stimuli for a task that is very close to a real-life work situation. It paves the way toward the efficient use of ERPs for MSM and brings us closer to the implementation of user adaptive systems in ecological settings.

## Author Contributions

Study conception and design: RR, SB, SC, AC. Acquisition of data: RR. Analysis and interpretation of data: RR, SB. Drafting of manuscript: RR. Critical revision: SB, SC, AC.

## Conflict of Interest Statement

The authors declare that the research was conducted in the absence of any commercial or financial relationships that could be construed as a potential conflict of interest.
